# A Nested Case-Control Study of Association between Metabolome and Hypertension Risk

**DOI:** 10.1155/2016/7646979

**Published:** 2016-03-29

**Authors:** Yongchen Hao, Ying Wang, Lu Xi, Guoqi Li, Fan Zhao, Yue Qi, Jing Liu, Dong Zhao

**Affiliations:** Department of Epidemiology, Beijing An Zhen Hospital, Capital Medical University, The Key Laboratory of Remodeling-Related Cardiovascular Diseases, Beijing Institute of Heart, Lung and Blood Vessel Diseases, Beijing 100029, China

## Abstract

We aimed to explore novel small metabolites that associated with hypertension risk in a population-based nested case-control study. Among 460 individuals with optimal blood pressure (<120/80 mmHg) at baseline, 55 progressed to hypertension during 5 years of follow-up. Twenty-nine cases of incident hypertension and 29 controls, matched for age, sex, and baseline systolic blood pressure, were included in this study. Serum metabolites were measured by gas chromatography-tandem mass spectrometry. *t*-test and logistic regression analysis were applied to investigate the association between metabolites and incident hypertension. Among the 241 metabolites identified in this study, baseline levels of 26 metabolites were significantly different between hypertension and control groups. After adjusting for body mass index, smoking, and drinking, 16 out of the 26 metabolites were still associated with hypertension risk including four amino acids. Amino acids were negatively associated with risk of future hypertension, with odds ratio (OR) ranging from 0.33 to 0.53. Two of these amino acids were essential amino acids including threonine and phenylalanine. Higher level of lyxose, a fermentation product of gut microbes, was associated with higher risk of hypertension. Our study identified multiple metabolites that associated with hypertension risk. These findings implied that low amino acid levels and gut microbiome might play an important role in the pathogenesis of hypertension.

## 1. Introduction

Hypertension is a key risk factor for cardiovascular diseases. It is estimated that 2.33 million cardiovascular deaths were attributable to increased blood pressure (BP) in China [1]. With the economic development and lifestyle changes in recent years, there has been a dramatic increase in the prevalence of hypertension in China [2]. Prevention of hypertension can help to reduce the public health burden of cardiovascular diseases [3]. It is critical to understand the mechanisms underlying elevated BP and develop reliable prevention strategies for populations at high risk of developing hypertension.

Hypertension is a metabolic disease and its pathophysiology is still unclear. Recent advances in metabolomic technologies have enhanced the feasibility of acquiring high-throughput profiles of a whole organism's metabolic status [4]. As these techniques allow assessment of large numbers of small metabolites that are substrates and products in metabolic pathways, metabolomics can particularly increase the understanding of the pathophysiology of metabolic diseases such as hypertension [5]. Although some studies have evaluated metabolomic differences in participants with and without hypertension [6–11], few have sought to identify baseline metabolites that predict future hypertension [12]. Metabolites identified in these studies that associated with hypertension were not well replicated. There are great metabolic differences between populations [8] and no study has explored the relationship between metabolites and risk of hypertension in Chinese population, which has the largest number of hypertensive patients [13]. Therefore, we aimed to explore small metabolites that independently associated with incident hypertension in a cohort based nested case-control study. In addition, we used pathway analysis to identify possible metabolic pathways implicated in the development of hypertension.

## 2. Materials and Methods

### 2.1. Study Participants

Participants were recruited from the Chinese Multiprovincial Cohort Study- (CMCS-) Beijing Project [14], part of the nationwide population-based CMCS study investigating the risk factors related to the incidence of cardiovascular diseases [15]. The flow chart of participant selection was presented in Fig. S1 in Supplementary Material available online at http://dx.doi.org/10.1155/2016/7646979. Of the 1478 participants who were free of hypertension and completed the baseline examination in 2002, 1133 took part in follow-up examination in 2007, with a follow-up rate of 76.7%. Among them, 460 were with optimal BP (<120/80 mmHg) at baseline. Fifty-five of these 460 participants developed hypertension and 208 participants were still with optimal BP in 2007. In this nested case-control study, 34 cases were randomly selected from the 55 participants who had optimal BP in 2002 and developed incident hypertension during follow-up. For each hypertensive case, we selected a control with optimal BP in both 2002 and 2007 and matched for gender, age (±3 years), and baseline systolic BP (±5 mmHg). Hypertension was defined as BP ≥ 140/90 mmHg and/or on antihypertensive therapy [16]. After excluding participants with more than 80% of metabolites below the detection limit or missing (five cases and five controls), 58 participants (29 cases and 29 controls) were eligible for final analysis. There was no significant difference in baseline characteristics between included participants and those excluded (Table S1).

All participants signed informed consent, and the Ethics Committee of Beijing An Zhen Hospital, Capital Medical University, reviewed and approved the protocols of this study.

### 2.2. Risk Factor Survey

The surveys in 2002 and 2007 were both conducted based on the WHO-MONICA (Monitoring of Trends and Determinants in Cardiovascular Disease) protocol for risk factor surveys. A standard questionnaire was designed to collect information on demographic characteristics, status of smoking and alcohol drinking, and personal medical history. Current smoking was defined as having smoked at least one cigarette per day in the past year. Drinking was defined as drinking at least once a week. Anthropometric measurements were recorded during physical examination. Body mass index (BMI) was calculated as weight in kilograms divided by height squared in meters. BP was measured in the right arm at a sitting position with a regular mercury sphygmomanometer after resting for at least 5 min. The mean value of two consecutive BP readings was used.

### 2.3. Laboratory Assays

Fasting total cholesterol (TC), triglyceride (TG), and fasting blood glucose (FBG) were determined by enzymatic methods. Low-density lipoprotein cholesterol (LDL-C) and high-density lipoprotein cholesterol (HDL-C) were measured by homogeneous assay (Daiichi, Tokyo, Japan). Overnight fasting venous blood samples were collected for laboratory measurements. To avoid the introduction of any analytical bias due to sample preparation, all samples were kept at room temperature to clot for 30 minutes and then put in ice box. Blood samples were centrifugated for 10 min (4000 ×g, 25°C) within three hours after blood collection. Serum was frozen at −80°C until execution of metabolomic analyses.

Metabolite profiles were measured using the gas chromatography-tandem mass spectrometry (GC/MS) method. Serum samples were thawed at room temperature for 15 min and vortex-mixed for 5 s. For GC/MS measurement, 300 *μ*L pure ethanol (HPLC grade: Sigma-Aldrich, St. Louis, MO, USA) was added to a 100 *μ*L serum sample in an Eppendorf tube. The mixture was vortex-mixed for 30 s and allowed to stand for 20 min at 4°C. After 80 *μ*L pure methanol (HPLC grade: Sigma-Aldrich) was added, the mixture was vortex-mixed for another 30 s and centrifuged at 4°C at 12000 rpm for 15 min. Then, 150 *μ*L of the supernatant was transferred to a screw vial and 10 *μ*L dichlorophen (0.02 mg/mL: Sigma-Aldrich) was added as the internal standard. The mixture was evaporated to dry under a stream of nitrogen gas (4°C). After 30 *μ*L methoxyamine pyridine hydrochloride (20 mg/mL) was added into the screw vial, the mixture was vortex-mixed for 30 s and oximated at 37°C for 90 min. We added 30 *μ*L* N,O*-bis(trimethylsilyl)trifluoroacetamide with 1% trimethylchlorosilane (Sigma-Aldrich) to each vial and left the mixture to react at 70°C for 60 min. The samples were allowed to stand for 15 min at room temperature before GC/MS analysis.

A 1 *μ*L aliquot of derivatized sample was injected in splitless mode into an Agilent 7890A GC system equipped with a 30 m × 0.25 mm × 0.25 *μ*m capillary column (Agilent J&W Scientific, Folsom, CA, USA). The injector temperature was set at 280°C and helium was used as carrier gas. The column temperature was initially kept at 80°C for 2 min, then increased from 80°C to 320°C at 10°C min^−1^, and held for 6 min. The column effluent was introduced into ion source of an Agilent 5975C mass detector. The ion source temperature was set at 230°C and the MS quadrupole temperature at 150°C [17]. Masses were acquired from 50 to 550* m/z*. For quality control, we performed metabolite profiling in the same quality control serum sample, enabling detection of temporal drift in instrument performance. Each of these injections into the mass spectrometer was generated from a distinct 10 *μ*L aliquot of pooled serum, extracted, and processed individually. The coefficients of variations (CVs) for each metabolite across a total of six replicates of quality control serum samples were calculated. Seventy-eight percent of the metabolites had CVs of less than or equal to 20%.

GC/MSD ChemStation Software (Agilent, Shanghai, China) was used for autoacquisition of GC total ion chromatograms and fragmentation patterns. Each compound had a fragmentation pattern comprising a series of split molecular ions; the mass charge ratios and their abundance were compared with a standard mass chromatogram in the National Institute of Standards and Technology (NIST) mass spectra library by the ChemStation Software. For each peak, the software generated a list of similarities comparing with every substance within the NIST library. Peaks with similarity index more than 70% were assigned compound names.

### 2.4. Statistical Analyses

Natural logarithm transformation was performed for continuous variables to minimize the skewedness of distribution if necessary. Metabolites with <50% BDL/missing observations were treated as continuous variables in the analyses; and metabolites with 50%–80% BDL/missing observations were treated as ordinal variables. A two-sample *t*-test or Wilcoxon rank-sum test was used for comparison of metabolite levels between hypertension and control groups. We also performed logistic regression analysis to investigate the association between metabolites and incident hypertension, adjusting for BMI, smoking, and drinking. A sample size of 58 (29 in hypertension group and 29 in control group) has a power of 0.88 to detect the difference between two groups, assuming the mean concentrations of 11.6 ± 2.57 and 9.65 ± 2.15 *μ*mol/L in hypertension and control groups, respectively [18], with a significance level of 0.05. A Bonferroni procedure was used to correct for multiple comparisons and a significance level of 2.07 × 10^−4^ (2-tailed) was considered for each individual test. Statistical analysis was performed with SAS 9.2 (SAS Institute Inc., Cary, NC, USA).

Metabolite set enrichment analysis was performed by MetaboAnalyst 2.0, a comprehensive, web-based tool designed for processing, analyzing, and interpreting metabolomic data [19]. Overrepresentation analysis was implemented using the hypergeometric test to evaluate whether a particular metabolite set was represented more than that expected by chance within a given compound list [19]. Metabolites with significant *P* values for hypertension risk were given as input in our study.

## 3. Results

### 3.1. Baseline Characteristics

Of the 1133 participants who were free of hypertension and were followed up for 5 years, 460 were with optimal BP at baseline. Among them, 55 developed hypertension during follow-up. Baseline characteristics of 29 new hypertensive cases and 29 controls included in this study are shown in Table 1. Age, gender, and systolic BP were matched variables. There were no significant differences in BMI, diastolic BP, FBG, TC, LDL-C, HDL-C, CRP, and creatinine at baseline between cases and controls.

### 3.2. Baseline Metabolite Levels and Risk of Hypertension

A total of 241 metabolites were identified in this study and the associations between baseline metabolite levels and risk of new onset hypertension were evaluated. Baseline levels of 26 metabolites were different between hypertension and control groups, including eight amino acids, seven carbohydrates, four carboxylic acids, three phenols, and four metabolites of other classes (Table 2). Five of these metabolites remained significant after Bonferroni correction including threonine (*P* = 1.78 × 10^−4^), talose (*P* = 9.01 × 10^−5^), lyxose (*P* = 4.26 × 10^−5^), methylmalonic acid (*P* = 2.37 × 10^−5^), and malonic acid (*P* = 1.24 × 10^−5^), and one with marginal significance (galactose, *P* = 5.71 × 10^−4^). Hypertension cases had lower baseline levels of amino acids than controls. After adjusting for BMI, smoking, and drinking, 16 out of the 26 metabolites were still significantly associated with hypertension risk and two of them were essential amino acids including threonine (odd ratio (OR): 0.33, 95% CI: 0.16–0.70, *P* = 1.78 × 10^−4^) and phenylalanine (OR: 0.49, 95% CI: 0.26–0.91, *P* = 1.12 × 10^−2^). A higher baseline level of lyxose, a fermentation product of gut microbes, was associated with higher risk of hypertension (OR: 2.88, 95% CI: 1.44–5.73, *P* = 4.26 × 10^−5^) (Table 2).

### 3.3. Relationship between Baseline Levels of Metabolites

**Figure 1:**
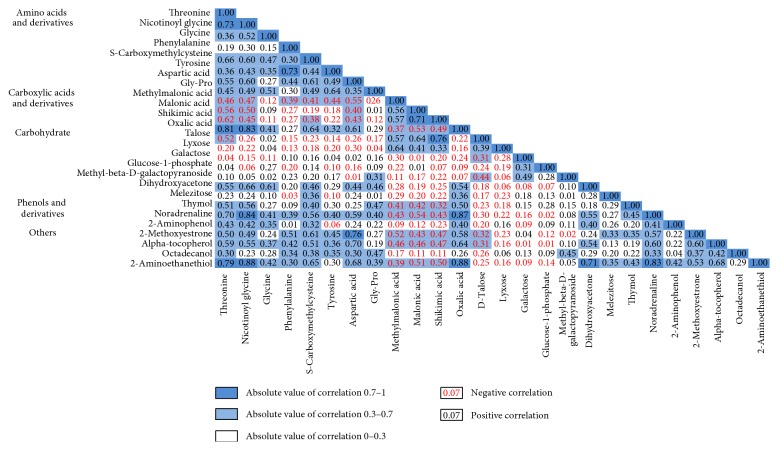
Correlation matrix for serum metabolite levels. This figure shows Spearman correlation coefficients for baseline levels of 26 metabolites that were different between hypertension and control groups. Correlation coefficients in red represent a negative correlation and in black represent a positive correlation.

We assessed correlations between baseline levels of 26 metabolites associated with hypertension risk. Amino acids were positively correlated with phenols and negatively correlated with carbohydrates and carboxylic acids (Figure 1). Phenylalanine was positively correlated with downstream metabolites within the phenylalanine and tyrosine metabolism pathway including tyrosine (*r* = 0.73) and norepinephrine (*r* = 0.39). Talose level was positively correlated with methylmalonic acid (*r* = 0.57) and malonic acid (*r* = 0.64) and negatively correlated with threonine (*r* = −0.52).

Pathway enrichment analysis was performed using the 26 metabolites associated with hypertension risk to identify novel pathways implicated in hypertension. Three metabolic pathways were identified, including metabolism of phenylalanine, tyrosine, and tryptophan biosynthesis (*P* = 1.92 × 10^−3^), aminoacyl-tRNA biosynthesis (*P* = 4.91 × 10^−3^), and nitrogen metabolism (*P* = 5.57 × 10^−3^) (Table 3). Of note, four out of five metabolites involved in these pathways were amino acids.

## 4. Discussion

Our study aimed to investigate human serum metabolites and hypertension risk in a well-defined nested case-control setting in a Chinese population. This nested case-control study examined metabolite profiles associated with the risk of hypertension. We identified a panel of metabolites whose baseline levels were associated with future development of hypertension. We found that participants who developed hypertension had reduced serum levels of amino acids, which implied that amino acids play an important role in development of hypertension. In addition, a gut microbial metabolite, lyxose, was associated with an elevated risk of hypertension.

It is worth noting that low levels of amino acids were associated with higher risk of hypertension in our study and two of them are essential amino acids (threonine and phenylalanine). Essential amino acids cannot be synthesized by the body, and levels of these amino acids in the body may mainly be decided by dietary protein intake. Recent studies suggest that inadequate intake of protein may lead to a shortage of essential amino acids and a subsequent elevation in BP [20, 21]. Evidence from randomized clinical trials indicates that administration of amino acids can improve endothelial function and decrease peripheral vascular resistance, resulting in decreased BP [22]. Amino acid metabolism may also regulate BP through insulin signaling, as it is essential to normal pancreatic *β*-cell function and insulin secretion [23]. In recent decades, the dietary pattern has changed rapidly in China. Data from the China Health and Nutrition Survey showed that the percentage of energy consumed from fat and protein (especially animal protein) had been increased in the past decades, while energy consumed from carbohydrate had decreased in China [24, 25]. However, protein intakes of majority of population were still below the amount recommended by dietary guidelines.

The effects of threonine and phenylalanine on hypertension are supported by findings from experimental studies. In Sprague-Dawley rats, threonine-deficient diets can induce a specific uncoupling of mitochondria [26] and reduce mitochondrial ATP production. Attenuated intracellular ATP content results in elevated BP by increased sympathetic nervous system activation, whereas augmented reactive oxygen production following mitochondrial dysfunction lowers the capacity of nitric oxide-dependent vascular relaxation [27, 28]. Phenylalanine intervention could exert an antihypertension effect on spontaneously hypertensive rats (SHR) [29]. The antihypertensive action of phenylalanine observed in SHR could be explained by its direct and specific antiproliferative effect on vascular smooth muscle cells [30]. Our study demonstrated that in humans depletion of phenylalanine can also influence metabolites in its downstream pathway. In our study, baseline phenylalanine was significantly depleted in patients with hypertension, and along with its depletion there was downregulation of many metabolites within the phenylalanine and tyrosine metabolic pathway (such as tyrosine and norepinephrine).

Our study illustrates the power of untargeted metabolic profiling to identify new metabolic pathways implicated in the development of hypertension. Three metabolic pathways were identified including phenylalanine, tyrosine, and tryptophan biosynthesis, aminoacyl-tRNA biosynthesis, and nitrogen metabolism pathways, which again emphasizes the importance of amino acid metabolism in the development of hypertension. The phenylalanine, tyrosine, and tryptophan biosynthesis pathway may take part in BP regulation through the antiproliferative effect of phenylalanine on vascular smooth muscle cells [30]. The nitrogen metabolism pathway can affect production of nitrogen compounds including nitric oxide which is evolved in BP regulation. A more comprehensive assessment of metabolites involved in these pathways in a larger sample is warranted and could shed further light on their mechanisms in BP regulation.

A novel finding of our study was that a higher baseline lyxose level was associated with a higher risk of hypertension. Lyxose is an aldopentose, a monosaccharide containing five carbon atoms including an aldehyde functional group. Lyxose is a key component of the bacterial cell wall [31] and is also a fermentation product of gut microbes which was reported as a potential biomarker of type 2 diabetes mellitus [32]. Human gut microbes can affect the amount of energy extracted from the diet and the risk of obesity, which in turn relates to BP [33]. Moreover, the ARIC study also identified a product of microbial fermentation, 4-hydroxyhippurate, which can take part in BP regulation through oxidative stress [12]. These findings may be associated with metabolic abnormalities of gut flora and demonstrate the important role of gut microbes in the development of hypertension. Diet pattern is known to modulate the composition of the gut microbiota. Also, products of gut microbial metabolism act as signaling molecules and influence the host's metabolism [34]. Supplementing the diet that stimulates the expansion of specific microbes to improve metabolic regulation can be a therapy for metabolic diseases [34]. Recent meta-analysis of randomized, controlled trials on consumption of probiotics revealed that probiotics are a potential supplement and dietary constituent to improve blood pressure and prevent or control hypertension [35].

This study has several strong points. All individuals were free of hypertension at the time the blood samples were collected and this enabled us to investigate the association between baseline metabolites and the risk of incident hypertension. We used a matched case-control design with extreme phenotype to maximize the efficiency of the study. We also matched cases and controls for age and gender as well as baseline systolic BP and adjusted for risk factors at baseline to minimize potential confounding contributions. In addition, we applied a pathway approach to highlight the key associations between metabolic measures and hypertension.

Our study has some potential limitations. First, this is an exploratory research with small sample size and without replications study. Therefore the candidate metabolites of interest from this study should be replicated in studies using greater sample sizes. Second, to what degree the results presented here are ethnically specific is unknown. Previous studies showed there were great metabolic differences between populations [8]. Whether candidate metabolites identified from this study have a role in the development of hypertension in other populations requires further investigation. In addition, we did not include other factors that might influence the regulation of blood pressure.

In conclusion, our study identified multiple metabolites that associated with risk of new onset hypertension. These findings implied that low amino acid levels and gut microbiome might play an important role in the pathogenesis of hypertension. Further investigation is required to test whether measurements might help identify metabolic candidates for interventions to reduce hypertension risk and to elucidate the biological mechanisms of BP regulation.

## Supplementary Material

Supplementary Table 1 contains baseline characteristics of participants included and not included in the study.
Supplementary Figure 1 shows flow chart of participant selection.

## Figures and Tables

**Table 1:** Baseline characteristic of study participants.

Characteristics	Individuals who developed hypertension (*n* = 29)	Individuals with optimal blood pressure (*n* = 29)	*P* ^*∗*^
Age (years)	52.1 ± 4.2	51.9 ± 4.1	0.84
Men (%)	44.8	41.4	0.79
BMI (kg/m^2^)	23.2 ± 2.6	24.0 ± 2.8	0.31
SBP (mmHg)	110.8 ± 6.6	110.2 ± 6.4	0.72
DBP (mmHg)	72.9 ± 4.1	72.4 ± 4.8	0.79
FBG (mg/dL)	83.9 ± 12.7	82.4 ± 7.9	0.60
TG (mg/dL)	84 (65, 106)	96 (64, 111)	0.64
TC (mg/dL)	202.9 ± 35.1	199.9 ± 30.0	0.73
LDL-C (mg/dL)	121.1 ± 30.3	116.5 ± 23.1	0.52
HDL-C (mg/dL)	56.6 ± 10.7	60.7 ± 15.9	0.26
CRP (mg/dL)	0.60 (0.30, 0.81)	0.68 (0.25, 1.17)	0.30
Creatinine (mg/dL)	0.99 ± 0.17	0.96 ± 0.18	0.54
Smoking (%)	20.7	10.3	0.47
Drinking (%)	13.8	6.9	0.67

BMI: body mass index; SBP: systolic blood pressure; DBP: diastolic blood pressure; FBG: fasting blood glucose; TG: triglycerides; TC: total cholesterol; LDL-C: low-density lipoprotein cholesterol; HDL-C: high-density lipoprotein cholesterol; CRP: C reactive protein. Data are expressed as a percent for categorical variables, as mean ± standard deviation for continuous variables in cases of normally distributed data, and as medians (interquartile ranges) otherwise. ^*∗*^ *P* values for the difference between two groups.

**Table 2:** Association of metabolites with incident hypertension.

Class	Metabolite	RT	Fold change	*P*	OR	*P*_adj_
Amino acids and derivatives	Threonine	21.87	0.12	1.46 × 10^−4^	0.33 (0.16–0.70)	1.78 × 10^−4^
	Nicotinoyl glycine	8.28	0.49	2.31 × 10^−2^	0.52 (0.28–0.96)	1.21 × 10^−2^
	Glycine	8.58	0.36	2.71 × 10^−2^	0.78 (0.44–1.38)	1.31 × 10^−1^
	Phenylalanine	13.02	0.70	3.44 × 10^−2^	0.49 (0.26–0.91)	1.12 × 10^−2^
	S-Carboxymethylcysteine	24.78	0.49	4.03 × 10^−2^	0.58 (0.32–1.05)	6.56 × 10^−2^
	Tyrosine	16.70	0.58	4.76 × 10^−2^	0.73 (0.42–1.29)	3.79 × 10^−1^
	Aspartic acid	21.22	0.55	4.92 × 10^−2^	0.53 (0.28–0.99)	2.38 × 10^−2^
	Gly-Pro	13.38	0.40	4.99 × 10^−2^	0.71 (0.4–1.23)	1.02 × 10^−2^

Carbohydrate	Talose	16.88	10.88	1.69 × 10^−6^	11.64 (3.39–39.96)	9.01 × 10^−5^
	Lyxose	14.47	2.29	1.24 × 10^−4^	2.88 (1.44–5.73)	4.26 × 10^−5^
	Galactose	16.49	0.22	2.81 × 10^−4^	0.34 (0.17–0.7)	5.71 × 10^−4^
	Glucose-1-phosphate	16.41	1.04 × 10^−6^	1.06 × 10^−3^	0.34 (0.16–0.71)	2.40 × 10^−3^
	Methyl-beta-D-galactopyranoside	14.32	0.37	6.73 × 10^−3^	0.28 (0.13–0.6)	1.20 × 10^−3^
	Dihydroxyacetone	7.06	0.50	3.12 × 10^−2^	0.62 (0.34–1.11)	9.84 × 10^−2^
	Melezitose	23.25	0.48	3.81 × 10^−2^	0.54 (0.29–1.01)	8.38 × 10^−2^

Carboxylic acids and derivatives	Methylmalonic acid	8.13	3.04	1.47 × 10^−7^	5.24 (2.1–13.03)	2.37 × 10^−5^
	Malonic acid	11.16	12.47	3.29 × 10^−5^	12.55 (3.4–46.39)	1.24 × 10^−4^
	Shikimic acid	14.33	5.56	5.85 × 10^−3^	5.06 (2.07–12.33)	3.00 × 10^−4^
	Oxalic acid	6.19	0.56	3.29 × 10^−2^	0.57 (0.31–1.02)	7.11 × 10^−2^

Phenols and derivatives	Thymol	14.17	0.31	4.14 × 10^−3^	0.43 (0.23–0.83)	6.00 × 10^−3^
	Noradrenaline	8.45	0.49	7.05 × 10^−3^	0.46 (0.24–0.87)	5.49 × 10^−3^
	2-Aminophenol	9.19	0.34	1.02 × 10^−2^	0.55 (0.29–1.01)	5.44 × 10^−2^

Others	2-Methoxyestrone	19.65	0.57	1.74 × 10^−2^	0.39 (0.2–0.76)	8.71 × 10^−3^
	Alpha-tocopherol	27.36	0.60	3.23 × 10^−2^	0.47 (0.25–0.89)	3.82 × 10^−2^
	Octadecanol	19.08	0.56	3.98 × 10^−2^	0.64 (0.36–1.14)	2.47 × 10^−1^
	2-Aminoethanethiol	6.08	0.59	4.26 × 10^−2^	0.59 (0.32–1.06)	9.38 × 10^−2^

RT: retention time; fold change: ratio of metabolite levels between case and control groups; OR: odds ratio; *P*_adj_: *P* value adjusted for BMI, smoking, drinking, creatinine and C reactive protein, and postmenopausal status.

**Table 3:** Pathways associated with risk of hypertension.

Pathway	Total	Expected	Hit	Metabolites	*P* ^*∗*^
Phenylalanine, tyrosine, and tryptophan biosynthesis	27	0.26	3	Phenylalanine, tyrosine, shikimic acid	1.92 × 10^−3^
Aminoacyl-tRNA biosynthesis	75	0.72	4	Phenylalanine, glycine, threonine, tyrosine	4.91 × 10^−3^
Nitrogen metabolism	39	0.37	3	Phenylalanine, tyrosine, glycine	5.57 × 10^−3^

Total: the total number of compounds in the pathway. Expected: the expected matched number from the 26 metabolites associated with hypertension. Hit: the actual matched number from the 26 metabolites associated with hypertension. ^*∗*^ *P*: *P* value calculated from enrichment analysis.

## References

[1] He J., Gu D., Chen J. (2009). Premature deaths attributable to blood pressure in China: a prospective cohort study. *The Lancet*.

[2] Chen X., Wei W., Zou S. (2014). Trends in the prevalence of hypertension in island and coastal areas of China: a systematic review with meta-analysis. *American Journal of Hypertension*.

[3] Law M. R., Morris J. K., Wald N. J. (2009). Use of blood pressure lowering drugs in the prevention of cardiovascular disease: meta-analysis of 147 randomised trials in the context of expectations from prospective epidemiological studies. *BMJ*.

[4] Nicholson J. K., Wilson I. D. (2003). Understanding ‘global’ systems biology: metabonomics and the continuum of metabolism. *Nature Reviews Drug Discovery*.

[5] Nikolic S. B., Sharman J. E., Adams M. J., Edwards L. M. (2014). Metabolomics in hypertension. *Journal of Hypertension*.

[6] Brindle J. T., Nicholson J. K., Schofield P. M., Grainger D. J., Holmes E. (2003). Application of chemometrics to 1H NMR spectroscopic data to investigate a relationship between human serum metabolic profiles and hypertension. *Analyst*.

[7] De Meyer T., Sinnaeve D., Van Gasse B. (2008). NMR-based characterization of metabolic alterations in hypertension using an adaptive, intelligent binning algorithm. *Analytical Chemistry*.

[8] Holmes E., Loo R. L., Stamler J. (2008). Human metabolic phenotype diversity and its association with diet and blood pressure. *Nature*.

[9] Li Y., Nie L., Jiang H. (2013). Metabonomics study of essential hypertension and its Chinese medicine subtypes by using gas chromatography-mass spectrometry and nuclear magnetic resonance spectroscopy. *Evidence-Based Complementary and Alternative Medicine*.

[10] Liu Y., Chen T., Qiu Y. (2011). An ultrasonication-assisted extraction and derivatization protocol for GC/TOFMS-based metabolite profiling. *Analytical and Bioanalytical Chemistry*.

[11] Cheng S., Rhee E. P., Larson M. G. (2012). Metabolite profiling identifies pathways associated with metabolic risk in humans. *Circulation*.

[12] Zheng Y., Yu B., Alexander D. (2013). Metabolomics and incident hypertension among blacks: the atherosclerosis risk in communities study. *Hypertension*.

[13] Kearney P. M., Whelton M., Reynolds K., Muntner P., Whelton P. K., He J. (2005). Global burden of hypertension: analysis of worldwide data. *The Lancet*.

[14] Liu J., Wang W., Qi Y. (2014). Association between the lipoprotein-associated phospholipase a2 activity and the progression of subclinical atherosclerosis. *Journal of Atherosclerosis and Thrombosis*.

[15] Liu J., Hong Y., D'Agostino R. B. (2004). Predictive value for the Chinese population of the Framingham CHD risk assessment tool compared with the Chinese Multi-provincial Cohort Study. *Journal of the American Medical Association*.

[16] Chobanian A. V., Bakris G. L., Black H. R. (2003). The seventh report of the joint national committee on prevention, detection, evaluation, and treatment of high blood pressure: the JNC 7 report. *The Journal of the American Medical Association*.

[17] Jiye A., Trygg J., Gullberg J. (2005). Extraction and GC/MS analysis of the human blood plasma metabolome. *Analytical Chemistry*.

[18] Lip G. Y. H., Edmunds E., Martin S. C., Jones A. F., Blann A. D., Beevers D. G. (2001). A pilot study of homocyst(e)ine levels in essential hypertension: relationship to von Willebrand factor, an index of endothelial damage. *American Journal of Hypertension*.

[19] Xia J., Mandal R., Sinelnikov I. V., Broadhurst D., Wishart D. S. (2012). Metaboanalyst 2.0—a comprehensive server for metabolomic data analysis. *Nucleic Acids Research*.

[20] Teunissen-Beekman K. F. M., van Baak M. A. (2013). The role of dietary protein in blood pressure regulation. *Current Opinion in Lipidology*.

[21] Altorf-van der Kuil W., Engberink M. F., Brink E. J. (2010). Dietary protein and blood pressure: a systematic review. *PLoS ONE*.

[22] Vasdev S., Stuckless J. (2010). Antihypertensive effects of dietary protein and its mechanism. *International Journal of Angiology*.

[23] Newsholme P., Brennan L., Rubi B., Maechler P. (2005). New insights into amino acid metabolism, *β*-cell function and diabetes. *Clinical Science*.

[24] Zhai F. Y., Du S. F., Wang Z. H., Zhang J. G., Du W. W., Popkin B. M. (2014). Dynamics of the Chinese diet and the role of urbanicity, 1991–2011. *Obesity Reviews*.

[25] Zhang B., Zhai F. Y., Du S. F., Popkin B. M. (2014). The China Health and Nutrition Survey, 1989–2011. *Obesity Reviews*.

[26] Ross-Inta C. M., Zhang Y.-F., Almendares A., Giulivi C. (2009). Threonine-deficient diets induced changes in hepatic bioenergetics. *American Journal of Physiology—Gastrointestinal and Liver Physiology*.

[27] Puddu P., Puddu G. M., Cravero E., De Pascalis S., Muscari A. (2007). The putative role of mitochondrial dysfunction in hypertension. *Clinical and Experimental Hypertension*.

[28] Postnov Y. V., Orlov S. N., Budnikov Y. Y., Doroschuk A. D., Postnov A. Y. (2007). Mitochondrial energy conversion disturbance with decrease in ATP production as a source of systemic arterial hypertension. *Pathophysiology*.

[29] Zhao G., Li Z., Gu T. (2001). Antihypertension and anti-cardiovascular remodeling by phenylalanine in spontaneously hypertensive rats: effectiveness and mechanisms. *Chinese Medical Journal*.

[30] Gao P. J., Zhu D. L., Zhan Y. M., Stepien O., Marche P., Zhao G. S. (1998). L-phenylalanine and smooth muscle cell proliferation from shr and wky rats. *Sheng Li Xue Bao*.

[31] Khoo K.-H., Suzuki R., Dell A. (1996). Chemistry of the lyxose-containing mycobacteriophage receptors of *Mycobacterium phlei/Mycobacterium smegmatis*. *Biochemistry*.

[32] Zhu Y., Cong W., Shen L. (2014). Fecal metabonomic study of a polysaccharide, MDG-1 from *Ophiopogon japonicus* on diabetic mice based on gas chromatography/time-of-flight mass spectrometry (GC TOF/MS). *Molecular BioSystems*.

[33] Ley R. E., Turnbaugh P. J., Klein S., Gordon J. I. (2006). Microbial ecology: human gut microbes associated with obesity. *Nature*.

[34] Tremaroli V., Bäckhed F. (2012). Functional interactions between the gut microbiota and host metabolism. *Nature*.

[35] Khalesi S., Sun J., Buys N., Jayasinghe R. (2014). Effect of probiotics on blood pressure: a systematic review and meta-analysis of randomized, controlled trials. *Hypertension*.

