# Supplementary material for: A Nested Case-Control Study of Association between Metabolome and Hypertension Risk
Source: Biomed Res Int. 2016 Mar 29;2016:7646979. doi: 10.1155/2016/7646979 (PMC4828541; doi:10.1155/2016/7646979)
Supplement: Supplementary file 1 — Supplementary Table 1 contains baseline characteristics of participants included and not included in the study. Supplementary Figure 1 shows flow chart of participant selection. [file 7646979.f1.pdf]

Table S1. Baseline characteristics of participants included and not included in the study

Characteristics	Cases			Controls		
	Included	Not included	*P	Included	Not included	*P
N	29	5	--	29	5	--
Age (years)	52.1±4.2	51.2±6.3	0.65	51.9±4.1	52.4±4.0	0.81
Men (%)	44.8	20.0	0.38	41.4	40.0	1.00
BMI (kg/m^2)	23.2±2.6	22.0±2.4	0.33	24.0±2.8	25.2±1.9	0.33
SBP (mmHg)	110.8±6.6	113.6±4.72	0.37	110.2±6.4	115.8±3.6	0.07
DBP (mmHg)	72.9±4.1	72.8±4.8	0.96	72.4±4.8	74.2±2.6	0.42
FBG (mg/dl)	83.9±12.7	80.4±9.4	0.56	82.4±7.9	77.6±9.2	0.23
TG(mg/dl)	84(65, 106)	91(89, 168)	0.26	96(64, 111)	187(90, 284)	0.20
TC(mg/dl)	202.9±35.1	194.4±47.5	0.64	199.9±30.0	208.2±60.7	0.78
LDL-C(mg/dl)	121.1±30.3	122.0±40.5	0.95	116.5±23.1	107.0±45.8	0.67
HDL-C(mg/dl)	56.6±10.7	56.0±8.5	0.90	60.7±15.9	56.2±18.8	0.58
Smoke (%)	20.7	0	0.56	10.3	40.0	0.15
Drink (%)	13.8	0	1.00	6.9	0	1.00

Abbreviations: BMI, body mass index; SBP, systolic blood pressure; DBP, diastolic blood pressure; FBG, fasting blood glucose; TG, triglycerides; TC, total cholesterol; LDL-C, low-density lipoprotein cholesterol; HDL-C, high-density lipoprotein cholesterol. Data are expressed as a percent for categorical variables, as mean±standard deviation for continuous variables in cases of normally distributed data, and as medians (interquartile ranges) otherwise.* *P* values were calculated between participants included and not included in the study.

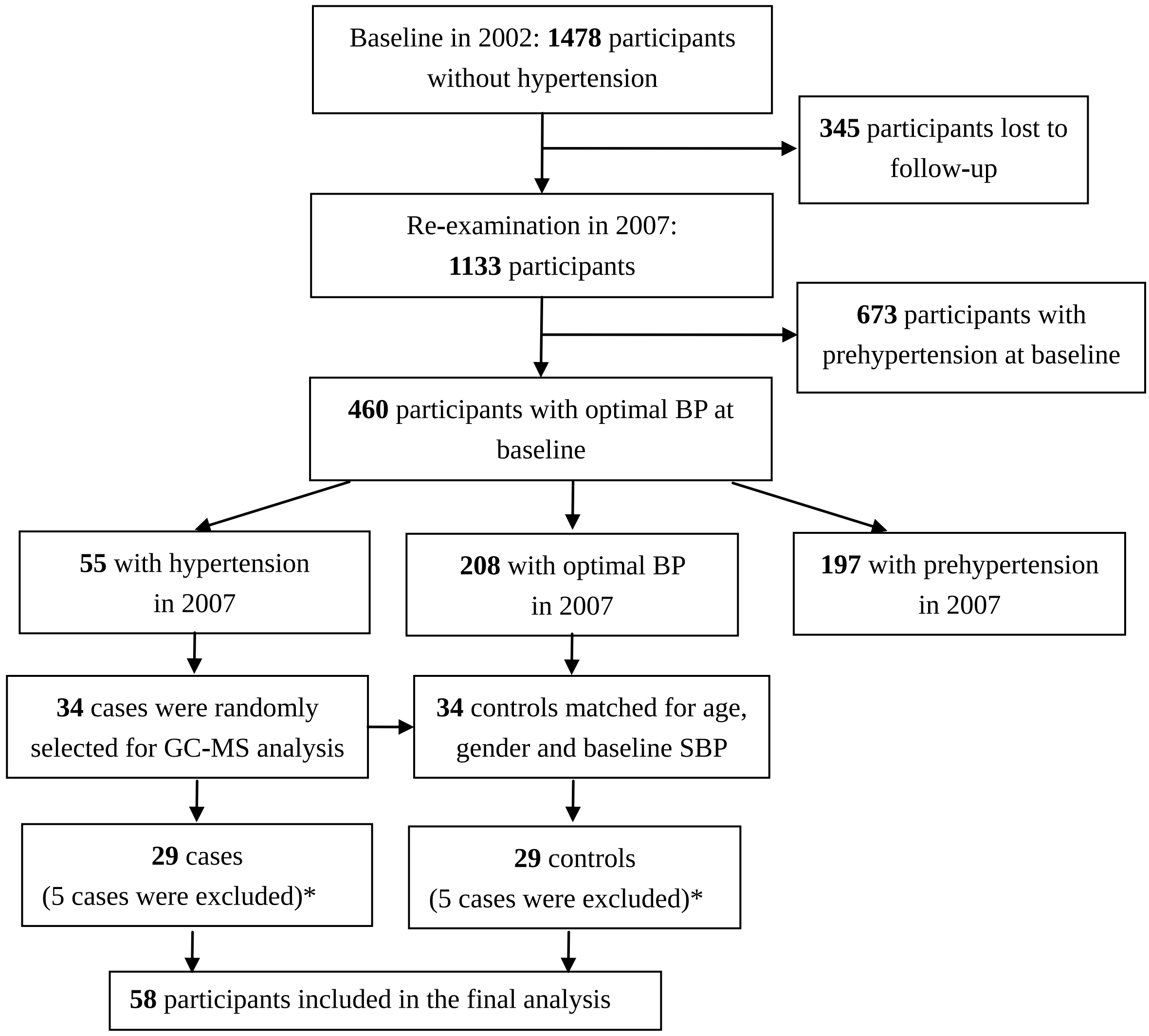

Figure S1 Flow chart of participant selection

* Participants with more than 80% of metabolites below the detection limit or missing
